# Effect of Supplementary Levels of Rumen-Protected Lysine and Methionine on Growth Performance, Carcass Traits, and Meat Quality in Feedlot Yaks (*Bos grunniens*)

**DOI:** 10.3390/ani11123384

**Published:** 2021-11-26

**Authors:** Zhiyuan Ma, Zhiwei Zhao, Hucheng Wang, Jianwei Zhou, Chengfu Zhang

**Affiliations:** 1 State Key Laboratory of Grassland Agro-Ecosystems, Key Laboratory of Grassland Livestock Industry Innovation, Ministry of Agriculture and Rural Affairs, College of Pastoral Agriculture Science and Technology, Lanzhou University, Lanzhou 730000, China; mazhy19@lzu.edu.cn (Z.M.); zhaozhw2018@lzu.edu.cn (Z.Z.); zhoujw@lzu.edu.cn (J.Z.); 2 Engineering Research Center of Grassland Industry, Ministry of Education, College of Pastoral Agriculture Science and Technology, Lanzhou University, Lanzhou 730000, China; 3 Institute of Animal Husbandry and Veterinary Medicine, Tibet Academy of Agricultural and Animal Husbandry Sciences, Lhasa 850000, China; tibetzcf@126.com

**Keywords:** feedlot yaks, rumen-protected lysine and methionine, growth performance, meat quality

## Abstract

**Simple Summary:**

Yak is an indigenous ruminant on the Qinghai-Tibetan Plateau and its meat is known as the “beef crown”. Traditionally, yak graze on the rangeland all year round and without any supplementation, and weight loss would occur in the cold season, which leads to rather low productive performance. In recent years, the strategy of intensive feedlot fattening was introduced to the yak raising industry. However, the results were not as encouraging as in the cattle steers, a fact that can be attributed to yak malnutrition due to differences in feed varieties supply and nutrients requirement between yaks and cattle. Consequently, this study was conducted to examine the effect of the first two limiting amino acids on growth performance, carcass traits, and meat quality in feedlot yaks. The animals were offered total mixed ration with supplementary four levels of rumen-protected lysine and methionine throughout the whole experiment. The results showed that the average daily gain, feed to gain ratio, meat color, drip loss, and shear force were improved when yaks were supplemented with rumen-protected amino acid while the medium level was most promising. These results indicated that supplementary rumen-protected amino acid improved the growth performance and meat quality characteristics of fattening yaks in feedlot.

**Abstract:**

Yak, a unique bovine species on the Qinghai-Tibetan Plateau, has provided pastoralists with the basic materials of production and life for thousands of years. Existing literature showed that yak meat is of high nutritional value for humans whereas the growth performance is relatively low. As it has been demonstrated, lysine and methionine are the two key limiting amino acids in ruminants. Therefore, this study was conducted to investigate the effect of supplementary levels of rumen-protected lysine (RPL) and methionine (RPM) on growth performance, carcass traits, and meat quality in feedlot yaks. Thirty-two male yaks, with initial body weight (IBW) of 237.5 ± 13.99 kg were randomly assigned to four dietary treatments (*n* = 8), including control group (CON; basal diet without adding rumen-protected amino acid (RPAA)), low RPAA supplementation group (Group Low; basal diet supplemented with RPL (30.0 g/animal/day) and RPM (7.5 g/animal/day)), medium RPAA supplementation group (Group Medium; basal diet supplemented with RPL (50.0 g/animal/day) and RPM (12.5 g/animal/day)), and high RPAA supplementation group (Group High; basal diet supplemented with RPL (70.0 g/animal/day) and RPM (17.5 g/animal/day)). The average daily gain was increased linearly (*p* < 0.001) and quadratically (*p* < 0.01) while feed to gain ratio was decreased linearly (*p* < 0.001) and quadratically (*p* < 0.001) with the increasing RPAA supplementation, and the average daily gain was higher while feed to gain ratio was lower in RPAA than CON (*p* < 0.001). Meat color L* values and b* were decreased linearly (*p* < 0.01) with the increase of RPAA supplementation, and b* was lower in RPAA than CON (*p* < 0.05). Meat color a* value was increased linearly (*p* < 0.05) with the increasing RPAA supplementation, and a* was higher in RPAA than CON (*p* < 0.05). The 24 h drip loss and shear force were decreased quadratically (*p* < 0.01) with the increasing RPAA supplementation, and drip loss and shear force values were lower in RPAA than CON (*p* < 0.05). The glycine concentration in *longissimus dorsi* (LD) increased linearly (*p* < 0.05) with the increasing RPAA supplementation. These results demonstrated that both the growth performance and meat quality characteristics were improved in feedlot yaks as an effect of the dietary supplementation with RPL and RPM, and the medium supplementary level is recommended, since it showed the most promising results.

## 1. Introduction

The Qinghai-Tibetan Plateau (QTP) is characterized by an extremely harsh environment, namely severe cold, hypoxia, and a short growing season [1,2]. Yak (*Bos grunniens*) is a specific indigenous ruminant that inhabits the QTP at the altitude between 3000 and 6000 m. Yak play a crucial role in the living, productive, and economic functions in this region. For instance, yak provide pastoralists with meat and milk for food, hair and hides for textiles, leather goods and clothing, and dung for fuel [3]. Under traditional management, yaks graze on natural pasture all year round and without any supplement. Consequently, with the seasonal variations of the biomass and nutrients in forages, the growth rate of yaks is dramatically affected, particularly in winter [4]. It was reported that grazing yaks would lose 25% of body weight during the cold season attributed to the feed deficiency, which significantly reduce the growth performance, and then damage the economic efficiency [5]. Based on this situation, the strategy of yak raising has been altered in recent years. A part of the finishing yaks would be gradually shifted from traditional grazing to semi-housing and housing management, which enhanced the productive performance and increased the profit. However, previous studies demonstrated that the nutrients requirement and metabolism in yaks were different with that in lowland ruminants [4,6], and there is still no feeding standard for fattening yaks up to now. Therefore, the feeding regimes are variable among yak feedlots and the effect of yak fattening is not as encouraging as in cattle steers.

In the Tibetan feedlots, ryegrass is the commonly used roughage while corn is the main ingredient for concentrate. It is reported that lysine (Lys) and methionine (Met) are the two first limiting essential amino acids (EAA) for growing beef cattle [7,8]. In addition, Lys and Met content in ryegrass (Lys: 0.41%; Met: 0.21%) and corn (Lys: 0.26%; Met: 0.19%) are below the requirement for protein synthesis in yaks [9]. Offering rumen-protected amino acids (RPAA) is an effective way of improving the amounts of metabolizable protein (MP) [10]. Existing literature indicated that supplementation of rumen-protected lysine (RPL) would increase the average daily gain (ADG) and feed conversion efficiency in cattle when fed corn-based diets [7,11]. Likewise, methionine is one of the sulfur-containing amino acids, which is critical for the health, growth, and meat quality of animals [8,12]. Recent studies observed that dietary N utilization was improved in beef cattle as a result of the supplementation with rumen-protected methionine (RPM) [13,14]. Furthermore, Liu et al. reported [15] that supplementation with RPL and RPM could affect rumen bacterial communities, which was positively associated with improved meat quality in yaks [16]. Cao et al. [17] demonstrated that the addition of RPL and RPM to the diet also had a positive effect on growth performance of yak calves. Similarly, supplementation of RPL and RPM increased dry matter intake (DMI), milk yield, and could influence rumen microbial populations, which in turn affected ruminal fermentation in lactating yaks [18]. These results suggested that RPL and RPM play an important role in the growth performance, nutrients metabolism, and physiological regulation in ruminants. As a result, it is reasonable to predict that Lys and Met supplementation is an effective approach to improve the growth performance and meat quality of yaks in Tibetan feedlots.

Yak meat is of high quality, since it is low in fat but high in protein content, and rich in minerals, essential amino acids, and polyunsaturated fatty acids as compared to commercial beef from lowland [19,20]. It was reported that more than 50% of the protein source for Tibetans is originated from yak meat [21]. However, there is still a critical discrepancy between the large demand for consumption of yak meat and the inefficient production in yak industry. To the best of our knowledge, the effect of supplementary RPL and RPM to feedlot yaks is scarcely studied. Therefore, we hypothesized that the appropriate supplementary level of RPL and RPM will improve growth performance, carcass traits, and meat quality of yaks. To test this hypothesis, four supplementary levels of RPL and RPM were used to evaluate above parameters in feedlot fattening yaks.

## 2. Materials and Methods

The animal use protocol and experiments procedures were approved by the Ethics Committee of the College of Pastoral Agriculture Science and Technology of Lanzhou University (Protocol number: LZU 201907030).

### 2.1. Location, Animals, and Diets 

The experiment was performed from August 2019 to November 2019 at the Tibet Tianmu Animal Husbandry Co., Ltd., of Naqu City (31°28.56′ N, 92°3.61′ E, and 4513 m altitude), Tibet, China. Thirty-two four-year-old male yaks with initial body weight (IBW) of 237.50 ± 13.99 kg were selected, and then were randomly assigned to 4 dietary treatments (*n* = 8). The treatments were control group (no supplementation of RPL and RPM, CON), and control diet supplemented with low (30.0 g RPL and 7.5 g RPM; Low), medium (50.0 g RPL and 12.5 g RPM; Medium), and high group (70.0 g RPL and 17.5 g RPM; High) level of RPL and RPM to each yak daily. The RPL (_L_-Lys ≥ 300 mg/g) and RPM (_DL_-Met ≥ 400 mg/g) products were purchased from DeHong Biotech Co., Ltd. (Hangzhou, China). The rumen bypass of RPL and RPM in the present study was ≥850 mg/g, and the small intestine absorption rate was ≥950 mg/g (data from supplier). There were no reports on the optimal levels of Lys and Met supplementation for feedlot yaks, and no specialized RPAA products are available for feedlot yaks; hence, the amounts of RPAA supplementation were according to the dosage recommended for beef cattle by the product instruction, and the RPAA was mixed and fed at a ratio of 3:1 (Lys: Met) [22].

The experimental yaks were housed in individual pens (5.5 × 3.5 m) during the whole experiment. The animals were fed total mixed ration ad libitum, which were offered twice daily at 10:00 a.m. and 17:00 p.m., and they had free access to drinking water. The whole experiment consisted of 2 periods, 7 days for adaptation period and 90 days for feeding trial. The adaptation period was used to allow yaks to become familiar with the diets, experimental procedures, and staff. Supplementary RPAA was mixed with one third of the morning feeding ration, which was in order to ensure the supplements could be completely consumed by yaks. The ingredients and chemical compositions of the experimental diets are presented in Table 1.

### 2.2. The Experimental Procedures and Sample Collection 

The diets offered and the refusals were recorded daily before morning feeding throughout the whole experiment. The samples of fresh diet and feed refusals were collected on days 15, 30, 45, 60, 75, and 90 of the feeding trial. The feed samples were mixed well for each sampling, and representative samples were used for chemical composition analysis. The body weight was weighed before the morning feeding. Daily feed intake, initial and final body weight (FBW) of yaks were recorded and ADG, DMI, and feed to gain ratio (F/G) were then calculated.

As the feeding trial completed, all of the yaks were transported to a nearby slaughter house, and fasted for 24 h before slaughter. The yaks were slaughtered following the usual practices of the Chinese beef industry in Tibet. Briefly, the yaks were stunned using a captive bolt pistol and then slaughtered by cutting the neck vessels. Then, the carcasses were washed, identified, and stored in a chilling chamber at 4 °C, where they remained for a 24 h period. Approximate 200 g meat samples of left *longissimus dorsi* (LD) were collected between the 12th and 13th ribs. The LD samples were subsequently divided into five sub-samples, and after epimysium removal, two sub-samples of LD were used for chemical composition and the pH_45min_ and pH_24h_ determination. The other three sub-samples were used to measure meat color, 24 h drip loss, and shear force, respectively.

### 2.3. Carcass Traits Measurements and Meat Quality Evaluation 

The hot carcass weight (HCW) was determined immediately after evisceration. The live weight before slaughter (LWBS) and HCW were measured to calculate the hot carcass dressing (HCD): HCD = (HCW/LWBS) × 100. The transversal area of the LD between the 12th and 13th ribs was defined as loin muscle area (LMA), which was determined using a parchment paper for each carcass. The marbling scores (MS) were subjectively evaluated as according to a standard image (marbling from 1 to 5, with 1 = no marbling and 5 = overly abundant marbling), and the average of values from different observers (*n* = 6) was used for each muscle sample.

The pH was determined using a portable pH meter (testo-205, Testo, Lenzkrich, Germany); the electrode was calibrated and an integrated temperature sensor to ensure temperature compensation and accurate measurement was used, then inserted into the muscle between the 12th and 13th ribs at the time of 45 min and 24 h post slaughtering. The meat color of the LD was evaluated after 30 min of blooming on day 1 of aging using the CIE L* a* b* system with an FRU WR-18 Chroma meter (Shenzhen Wave Optoelectronics Technology Co., Ltd., Shenzhen, China) (with a 10° view angle, D65 illuminant, and 8 mm aperture with a closed cone). Measurements of lightness (L*), redness (a*), and yellowness (b*) at three randomly selected points were recorded for each sample.

The water-holding capacity (WHC) of LD was determined by drip loss, which was followed the method of Honikel [23]. Briefly, three muscle cores were prepared using string, separately weighed, suspended without contact to plastic bags, and kept at 4 °C. After 24 h, the sample was removed from the bag, dried on absorbent paper, and reweighed, and drip loss was calculated as the weight differences. Shear force was measured as the following procedures: the epimysium of LD was removed and cooked in 80 °C water with an internal temperature of 70 °C, and cooled to room temperature for measuring shear force. Six cylindrical cores (1.27 cm diameter round sampler) of the cooked LD samples were cut perpendicular to the fiber orientation of the muscle using a meat tenderness tester (RH-N50, Guangzhou Runhu Instruments Co., Ltd., Guangzhou, China). The average peak shear force, expressed in newtons (N), was recorded.

### 2.4. Laboratory Analyses

Feed and orts samples were dried at 55 °C for 72 h and ground to pass through a 1 mm screen. The dry matter (DM) (method 934.01), calcium (method 927.02), and phosphorus (method 965.17) content were determined using an analytical method provided by the Association of Official Agricultural Chemists (AOAC) [24]. The N contents of the feed were determined using the micro-Kjeldahl N method, and crude protein (CP) was calculated as N × 6.25. The neutral detergent fiber (NDF, assayed without heatstable amylase and expressed inclusive of residual ash) and acid detergent fiber (ADF, expressed inclusive of residual ash) of feed was measured with a fiber analyzer (ANKOM 2000; ANKOM Technology, Macedon, NY, USA) followed the methods of Van Soest et al. [25]. 

Total amino acids (TAAs) in muscle firstly following hydrolysis, and then free amino acids (AAs) were analyzed with a high-performance liquid chromatography (HPLC) (Waters-e2695, Boston, MA, USA). Total amino acids were measured as follows: the LD samples (200 mg) were hydrolyzed using 10 mL HCL (6 mol/L) in hydrolytic tubes and kept in a thermostatic drier box for 4 h at 145 °C. The hydrolysates were transferred and diluted into 50 mL using volumetric flasks, and then with 10 uL diluted hydrolysates and 10 μL standard solution were mixed, subsequently vacuum dried (5 min), and 20 μL derivatization buffer solution accurately added (pH = 9.0). This mixed solution was blended well, and 20 μL derivatization reagent was added (1%, 2,4-nitrofluorobenzene acetonitrile), blended well again and sealed with parafilm, derivatized in an oven at 64 °C for 30 min, after cooling, 160 μL balance buffer solution was added, and the final solution was used for AA determination with HPLC.

### 2.5. Statistical Analyses 

The normality of all data was tested using the Shapiro–Wilk test and showed a normal distribution. Student’s *t*-test was used to assess the effect of the control treatment vs. rumen-protected amino acid groups, and an orthogonal contrast was used to evaluate the linear and quadratic response to the supplementary level of RPAA (*p* ≤ 0.05). All data was assessed by an analysis of variance using the General Linear Model procedure in SPSS v22.0 (IBM SPSS Statistics, SPSS Inc., Chicago, IL, USA), using the following model:

Yij = μ + β_1_X_i_ + β_2_X_i_^2^ + εij

where Yij = observation of the repetition j on diet i; μ = general coefficient; β_1_ = linear regression coefficient of the variable observed depending on the level; β_2_ = quadratic regression coefficient of the variable observed depending on the level; X_i_ = independent variables (blend of RPAA levels); Εij = residual error. In all statistical analyses, the diet was considered a fixed effect, and the yaks considered a random effect. The mean and standard error of the mean (SEM) were calculated for each variable. Statistical differences between the treatment were assessed using Duncan’s Test (*p* ≤ 0.05). 

## 3. Results

### 3.1. Feed Intakes and Body Weight Changes

The FBW and DMI were not affected (*p* > 0.05; Table 2) by supplementary levels of rumen-protected amino acids, and there was no difference (*p* > 0.05) observed between control and RPAA group in these two parameters. However, ADG was increased linearly (*p* < 0.001) and quadratically (*p* < 0.01) while F/G was decreased linearly (*p* < 0.001) and quadratically (*p* < 0.001) with the increasing RPAA supplementation, and ADG was higher while F/G was lower in RPAA than CON (*p* < 0.001). The greatest ADG and lowest F/G was detected in the medium supplementation group.

### 3.2. Carcass Characteristics

Carcass traits of feedlot yaks were determined and the results are shown in Table 3. The LWBS, HCW, HCD, LMA, and MS were not affected (*p* > 0.05) by the increasing levels of RPAA supplementation, and there was no difference (*p* > 0.05) observed between CON and RPAA as well.

### 3.3. Meat Quality

The meat pH at 45 min and 24 h were not affected (*p* > 0.05; Table 4) by supplementary level of rumen-protected amino acids, and also there was no difference (*p* > 0.05) observed between CON and RPAA. Meat color L* and b* were decreased linearly (*p* < 0.01) with the increasing RPAA supplementation, and b* was lower in RPAA than CON (*p* < 0.05). However, meat color a* was increased linearly (*p* < 0.05) with increasing RPAA supplementation, and a* was higher in RPAA than CON (*p* < 0.05). The 24 h drip loss and shear force both were decreased quadratically (*p* < 0.01) with the increasing RPAA supplementation, and drip loss and shear force were lower in RPAA than CON (*p* < 0.05). The lowest drip loss was observed in medium supplementation group while shear force was lower (*p* < 0.05) in low and medium groups as compared to CON and high groups.

### 3.4. Amino Acids Profiles

The AA profiles of LD are shown in Table 5, and there was no difference (*p* > 0.05) in the total content of AA (amino acids), EAA (essential amino acids), or NEAA (non-essential amino acids) between control and RPAA, and they also were not affected (*p* > 0.05) by the supplementary level of rumen-protected amino acids. All of the individual amino acids were not affected (*p* > 0.05) by rumen-protected amino acids supplementation except for glycine, which was increased linearly (*p* < 0.05) with the increasing of RPAA supplementation.

## 4. Discussion

### 4.1. Effect of RPAA Supplementation on Growth Performance and Carcass Traits 

Growth performance is influenced by the nutrient intakes and utilization efficiency of farm animals, which is highly associated with the economic profit. In ruminants, both the dietary protein and microbial protein that bypassed the rumen are calculated as the MP, which is hydrolyzed into peptides and amino acids, and then absorbed in the small intestine [26]. Either deficiency or imbalance of the amino acids profiles in MP would inhibit the growth performance and feed conversion efficiency of livestock [27]. In the present study, although there was no difference of FBW and LWBS between control and RPAA, the ADG of yaks was increased while F/G was decreased with increased of supplementary RPL and RPM. As mentioned before, the lysine and methionine in ryegrass and concentrate were relatively low, which indicated the inadequacy of these two amino acids offered to fattening yaks in Tibetan feedlots. In addition, the optimal supplementary effect was observed at the medium level, which was probably close to the ideal amino acid profile in yaks. This also suggests that low and high levels of RPAA supplementation are inefficient. However, the results in previous literature that supplemented with PRL and RPM were inconsistent. Xue et al. [11] reported that RPL supplementation did not alter DMI in growing cattle offered the maize stalk silage/maize grain-based diets, but increased ADG and feed efficiency and the optimal effect was observed at the dosage of 10 g/d RPL. More recently, Cao et al. [17] observed that supplementation with RPL and RPM could improve the chest girth, body index, FBW, ADG, and fatness index. Zhao et al. [18] reported supplementation of 50 g/d RPM increased DMI of lactating yaks. Similarly, Klemesrud et al. [28] observed supplementary RPM and RPL improved ADG in growing steers when fed with sorghum silage and round corn cob based diets. Nevertheless, the ADG and feed conversion efficiency in finishing cattle were not affected by supplementation of RPL when offered corn product-based diets [29]. Overall, these discrepant results of RPL and RPM supplementation was probably due to the differences in supplementary dosage, animal physiological status, and dietary composition.

The carcass traits, including HCW, HCD, LMA, and MS were not affected by supplementary RPL and RPM in feedlot yaks, which was in agreement with the study in crossbred beef cattle [30]. Moreover, previous studies in steers also observed that there was no effect on carcass traits when supplemented with RPL [7,29]. In contrast, Teixeira et al. [10] reported that supplementary RPL increased longissimus muscle area and improved carcass leanness rate and claimed that supplementation of RPL increased protein accretion and then facilitated muscle synthesis. Likewise, several studies in pigs also found that supplementary lysine would increase loin eye area and decrease fat tissue proportion as compared to the non-supplemented groups [31,32]. These aforementioned results indicated that the effect of rumen-protected amino acids was variable, which was dependent on animal species, types of amino acids, and feeding background [4,6]. In the present study, the HCD and MS were 48.72–51.01% and 2.38–3.25, respectively, which was lower than that of cattle steers reported in previous studies, but within the ranges in growing yaks [33]. 

### 4.2. Effect of RPAA Supplementation on Meat Quality

With the development of society and economy of human beings, high-quality, healthy, and safe meat is becoming more and more popular. The color of meat, a direct indicator of freshness, is remarkably influenced by the purchased decision of consumers rather than any other meat quality factors. In general, the color of fresh meat should be bright red or pink. The L* of meat color increases with ageing of the meat sample, which is related to the depth of mitochondrial respiration and oxygen penetration into the exposed muscle surface that remains in the muscle after slaughter [34]. The a* reflects the freshness of the muscle, which is affected by the state of the myoglobin. In addition, as storage time increases, the metabolism of parasitic bacteria on the surface of the muscle produces hydrogen sulphide and oxygen, which combine with myoglobin to form myoglobin sulphide, which increases the flesh color of the muscle b* and reduces the quality of the muscle [35]. In the present study, as compared to control group, meat L^*^ and b^*^ values of medium and high RPAA supplementary groups were decreased, whereas meat a^*^ value was increased in low and high RPAA supplementary groups. These results suggest that RPAA supplementation in yak fattening systems may result in changes in beef chromatic attributes. Tenderness is an index of taste, which is negatively related to shear force. Shear force is mainly affected by the thermal stability of muscle, the structure of collagen, and myofibril protein in meat [36]. Shear force is closely related to intramuscular fat [37]. The results of present study showed that low and medium supplementary RPL and RPM decreased the shear forces compared to the control group, which indicated that decomposition and accretion of protein and fat in yaks were probably altered. In contrast, a previous study suggested that the meat color of L* and a*, pH, and tenderness in steers were not affected by RPL supplementation but had a quadratic effect on meat b* [30]. Additionally, meat quality and carcass yield are closely related to the amount of potential capacity to retain moisture water after slaughter. Drip loss is a measure of water-weight loss because of gravimetric forces. This metric is able to provide insight into WHC, and consequentially it is valuable to evaluate meat quality [38]. The present results showed that medium supplementary RPAA decreased the 24 h drip loss in LD as compared to the control group, which indicated that the supplementation of RPL and RPM would improve WHC of yak meat. 

### 4.3. Effect of RPAA Supplementation on Amino Acids Profile

The amino acids profile is considered as an important indicator of meat quality, especially in the food science and nutrition area [39]. Up to now, there are still few reports about the effect of supplementation of RPL and RPM on amino acids profile in yak meat. Amino acids content and individual fractions in muscle are positively correlated with nutrient values, which would then influence meat odor traits, such as taste and fragrance [40]. In the present study, supplementation of RPL and RPM did not affect total and individual amino acids apart from glycine, a finding that is in agreement with the results in cattle when supplemented with RPL [30]. Meanwhile, glycine is one of the sweet amino acids [41], and glycine concentration was increased with RPL and RPM supplementation, which indicated the promotion of meat flavor in RPAA yaks. In addition, the amino acid composition of yak meat is very close to the requirement of the human organism. In the present study, 17 amino acids were detected in yak meat, and EAA/TAA ranged between 42.61% and 42.74%, all were greater than 40%. This result indicated that the amino acid score was close to the ideal amino acid model proposed by FAO/WHO (Food and Agriculture Organization of the United Nations; World Health Organization). Therefore, yak meat is of high quality for protein source, and supplementary RPL and RPM to yaks would improve the sweet traits in muscle. However, to our knowledge, the underlying cause of the effect of supplementation of RPL and RPM on the AA profiles in yak meat still remains unclear, and further investigation is required in future. In addition, exploring the appropriate ratio of lysine and methionine supplementation for yaks is something that should be considered in future research.

## 5. Conclusions

The ADG and redness were increased whereas lightness, yellowness, drip loss, and tenderness were decreased as yaks were supplemented with RPAA, and the medium supplementary level of RPL and RPM revealed the most promising effect. It was concluded that the growth performance and meat quality would be improved by supplementary RPAA, and the recommend supplementations for rumen bypassed lysine and methionine are 15.0 and 5.0 g per day for each fattening yak, respectively.

## Figures and Tables

**Table 1:** Ingredient and chemical composition of the experimental diets.

Item	Content
Ingredients, g/kg DM ^1^	
Ryegrass hay	600
Corn	300
Wheat bran	15.0
Cottonseed meal	35.0
Rapeseed meal	35.0
CaHPO_4_	0.50
Limestone	4.50
NaHCO_3_	5.0
NaCl	5.0
Chemical composition, g/kg DM	
Dry matter	860
Metabolizable energy ^2^ (MJ/kg)	94.4
Crude protein	110
Neutral detergent fiber	404
Acid detergent fiber	266
Calcium	4.50
Phosphorus	2.80
Lysine ^3^	2.90
Methionine ^3^	1.90

^1^ DM, dry matter. ^2^ Nutrient levels were measured values except for metabolizable energy. ^3^ The supplementary of RPL and RPM was not included.

**Table 2:** Effect of supplementary levels of rumen-protected amino acid on growth performance in feedlot yaks.

Item	Diets ^1^					*p*-Value		
	CON	Low	Medium	High	SEM	Linear	Quadratic	CON vs. RPAA ^2^
IBW, kg	238.75	237.50	237.86	236.25	10.793	0.944	0.994	0.944
FBW, kg	268.13	271.88	280.71	274.38	10.349	0.776	0.819	0.982
DMI, kg/d	4.04	4.17	4.31	4.19	0.046	0.136	0.161	0.205
ADG, g/d	326.39 ^c^	381.94 ^b^	476.19 ^a^	423.61 ^ab^	13.242	<0.001	<0.01	<0.001
F/G	12.38 ^a^	10.91 ^b^	9.06 ^d^	9.88 ^c^	0.332	<0.001	<0.001	<0.001

^1^ CON, control; Low, control + 30.0 g RPL and 7.50 g RPM per yak per day; Medium, control + 50.0 g RPL and 12.50 g RPM per yak per day; High, control + 70.0 g RPL and 17.50 g RPM per yak per day. ^2^ Rumen-protected amino acid. a, b, c, d: indicate statistical differences in the same row (*p* < 0.05). Abbreviations: IBW, initial body weight; FBW, final body weight; ADG, average daily gain; DMI, dry matter intake; F/G, the feed to gain ratio.

**Table 3:** Effect of supplementary levels of rumen-protected amino acid on carcass traits in feedlot yaks.

Item	Diets ^1^					*p*-Value		
	CON	Low	Medium	High	SEM	Linear	Quadratic	CON vs. RPAA ^2^
LWBS, kg	261.25	265.00	273.75	267.50	7.070	0.700	0.754	0.950
HCW, kg	127.72	130.25	139.50	132.38	3.475	0.494	0.524	0.706
HCD, %	48.72	49.17	51.01	49.49	0.372	0.188	0.166	0.139
LMA, cm^2^	39.44	42.47	43.63	42.05	1.882	0.634	0.585	0.470
MS ^3^	3.13	3.13	2.38	3.25	0.207	0.844	0.314	0.677

^1^ CON, control; Low, control + 30.0 g RPL and 7.50 g RPM per yak per day; Medium, control + 50.0 g RPL and 12.50 g RPM per yak per day; High, control + 70.0 g RPL and 17.50 g RPM per yak per day. ^2^ Rumen-protected amino acid. ^3^ The marbling score was subjectively evaluated (marbling from 1 to 5 with 1 = devoid and 5 = overly abundant). Abbreviations: LWBS, live weight before slaughter; HCW, hot carcass weight; HCD, hot carcass dressing; LMA, loin muscle area; MS, marbling score.

**Table 4:** Effect of supplementary levels of rumen-protected amino acid on organoleptic quality of *longissimus dorsi* in feedlot yaks.

Item	Diets ^1^					*p*-Value		
	CON	Low	Medium	High	SEM	Linear	Quadratic	CON vs. RPAA ^2^
pH_45min_	6.63	6.73	6.68	6.80	0.061	0.436	0.911	0.470
pH_24h_	5.51	5.44	5.31	5.51	0.045	0.744	0.153	0.404
Lightness, L*	32.14 ^a^	32.12 ^a^	27.60 ^b^	27.03 ^b^	0.844	<0.01	0.832	0.100
Redness, a*	8.91 ^b^	10.62 ^a^	9.95 ^ab^	11.05 ^a^	0.314	0.030	0.567	0.018
Yellowness, b*	6.51 ^a^	6.39 ^a^	4.72 ^b^	4.54 ^b^	0.263	<0.001	0.918	0.028
Drip loss, %	5.95 ^a^	5.23 ^ab^	4.66 ^b^	5.67 ^a^	0.185	0.276	<0.01	0.028
Shear force, N	64.68 ^a^	47.92 ^b^	53.36 ^b^	64.20 ^a^	2.418	0.779	<0.001	<0.01

^1^ CON, control; Low, control + 30.0 g RPL and 7.50 g RPM per yak per day; Medium, control + 50.0 g RPL and 12.50 g RPM per yak per day; High, control + 70.0 g RPL and 17.50 g RPM per yak per day. ^2^ Rumen-protected amino acid. a, b: indicate statistical differences in the same row (*p* < 0.05).

**Table 5:** Effect of supplementary levels of rumen-protected amino acid on amino acids content of *longissimus dorsi* in feedlot yaks (g/100 g of muscle).

Item	Diets ^1^					*p*-Value		
	CON	Low	Medium	High	SEM	Linear	Quadratic	CON vs. RPAA ^2^
EAA								
Isoleucine	4.56	4.55	4.60	4.57	0.072	0.835	0.947	0.996
Leucine	5.84	5.86	5.88	5.84	0.013	0.844	0.390	0.748
Lysine	7.30	7.33	7.36	7.30	0.025	0.929	0.485	0.889
Methionine	1.73	1.74	1.73	1.72	0.021	0.845	0.814	0.989
Phenylalanine	3.85	3.84	3.86	3.84	0.033	0.978	0.884	0.995
Threonine	2.66	2.65	2.67	2.64	0.031	0.913	0.911	0.991
Valine	6.51	6.55	6.53	6.59	0.057	0.707	0.951	0.974
NEAA								
Alanine	4.54	4.62	4.57	4.56	0.033	0.985	0.578	0.882
Arginine	4.88	4.91	4.91	4.93	0.024	0.558	0.954	0.937
Histidine	2.86	2.97	2.91	2.90	0.037	0.882	0.502	0.848
Aspartic acid	7.01	7.03	6.98	6.98	0.026	0.598	0.847	0.921
Glutamic acid	11.74	11.78	11.78	11.74	0.022	0.989	0.512	0.922
Glycine	4.85 ^ab^	4.78 ^b^	4.87 ^ab^	5.02 ^a^	0.036	0.046	0.086	0.084
Serine	1.94	1.97	2.02	1.96	0.013	0.348	0.089	0.170
Tyrosine	2.09	2.09	2.10	2.08	0.017	0.898	0.839	0.992
Proline	3.30	3.32	3.36	3.44	0.010	0.077	0.339	0.212
Cystine	0.24	0.27	0.21	0.25	0.015	0.779	0.917	0.609
Total EAAs	32.45	32.52	32.64	32.50	0.172	0.891	0.803	0.989
Total NEAAs	43.47	43.74	43.71	43.74	0.145	0.576	0.755	0.913
Total AAs	75.93	76.27	76.35	76.27	0.293	0.723	0.764	0.970
EAA/TAA	42.74	42.65	42.74	42.61	0.093	0.751	0.928	0.955
EAA/NEAA	75.93	75.80	74.14	72.92	0.601	0.061	0.636	0.242

^1^ CON—control; Low—control + supplemental RPL and RPM (30.0 and 7.50 g/yak daily, respectively); Medium—control + supplemental RPL and RPM (50.0 and 12.50 g/yak daily, respectively); High—control + supplemental RPL and RPM (70.0 and 17.50 g/yak daily, respectively). ^2^ Rumen-protected amino acid. a, b: indicate statistical differences in the same row (*p* < 0.05). Abbreviations: EAAs, essential amino acids; NEAAs, non-essential amino acids.

## Data Availability

Data sharing is not applicable.
